# Study of Forward and Backward Modes in Double-Sided Dielectric-Filled Corrugated Waveguides

**DOI:** 10.3390/s21186293

**Published:** 2021-09-20

**Authors:** Pilar Castillo-Tapia, Francisco Mesa, Alexander Yakovlev, Guido Valerio, Oscar Quevedo-Teruel

**Affiliations:** 1 Division of Electromagnetic Engineering, KTH Royal Institute of Technology, 114 28 Stockholm, Sweden; oscarqt@kth.se; 2 Department of Applied Physics 1, E.T.S. de Ingeniería Informática, University of Seville, 41012 Seville, Spain; mesa@us.es; 3 Department of Electrical and Computer Engineering, The University of Mississippi, Oxford, MS 38677, USA; yakovlev@olemiss.edu; 4 Laboratoire de Génie Electrique et Electronique de Paris, CNRS, Sorbonne Université, 75252 Paris, France; guido.valerio@sorbonne-universite.fr; 5 Laboratoire de Gènie Electrique et Electronique de Paris, CNRS, CentraleSupèlec, Universitè Paris-Saclay, 91192 Paris, France

**Keywords:** corrugated waveguide, glide symmetry, higher symmetries, Bloch analysis

## Abstract

This work studies the propagation characteristics of a rectangular waveguide with aligned/misaligned double-sided dielectric-filled metallic corrugations. Two modes are found to propagate in the proposed double-sided configuration below the hollow-waveguide cutoff frequency: a quasi-resonant mode and a backward mode. This is in contrast to the single-sided configuration, which only allows for backward propagation. Moreover, the double-sided configuration can be of interest for waveguide miniaturization on account of the broader band of its backward mode. The width of the stopband between the quasi-resonant and backward modes can be controlled by the misalignment of the top and bottom corrugations, being null for the glide-symmetric case. The previous study is complemented with numerical results showing the impact of the height of the corrugations, as well as the filling dielectric permittivity, on the bandwidth and location of the appearing negative-effective-permeability band. The multi-modal transmission-matrix method has also been employed to estimate the rejection level and material losses in the structure and to determine which port modes are associated with the quasi-resonant and backward modes. Finally, it is shown that glide symmetry can advantageously be used to reduce the dispersion and broadens the operating band of the modes.

## 1. Introduction

Rectangular metallic waveguides are among the first employed microwave transmission systems [1]. Hollow waveguides have been broadly used, mainly because of their low propagation losses and high-power capabilities. However, their frequency cut-off characteristics and bulkiness limit their use in some applications. In order to overcome these drawbacks, several studies adding metasurfaces have been conducted in order to allow for backward modes, which can propagate below the cut-off frequency [2,3,4,5]. In [6,7,8,9], it was demonstrated that a hollow waveguide with dielectric-filled periodic corrugations on the bottom wall allows the propagation of backward modes. Among other possible applications, this characteristic was advantageously employed in [6,7] to build slot-array antennas. Corrugated waveguides have also been proposed for sensing electric and magnetic fields [10], as well as temperature in the THz regime [11]. Here, we propose a waveguide with double-sided corrugations in order to increase propagation bandwidth of the backward modes. Moreover, we show that, depending on the design of the waveguide, it is possible to achieve an almost continuous propagation below and above the cut-off frequency of the hollow metallic waveguide. This advantage will allow for the use of more compact waveguides in millimetre wave systems. We also study the properties when these corrugations are misaligned, including the glide-symmetric configuration.

Glide symmetry is one of the so-called higher symmetries characterized by keeping invariant the periodic structure after reflection and translation of one-half the period [12]. The interest on glide symmetry has been recently boosted since it provides several interesting electromagnetic properties [13]. For example, it has been found that glide symmetry reduces the dispersion of the first propagating mode in metasurfaces, as well as increases its equivalent refractive index [14]. These properties are useful in the design of lenses, such as the Luneburg [15] and Maxwell fish-eye lens [16], and low-dispersive leaky-wave antennas [17]. Another useful property comes from its ability to increase the range of anisotropy of periodic structures. This feature can be used to compress the size of lens antennas [18,19]. Glide symmetry was also proposed to produce composite magnetic and electric materials, having a significant advantage with respect to conventional periodic structures to reduce the reflections for highly dense dielectric materials [20]. Finally, it was also shown that glide symmetry increases the frequency bandwidth of rejection of electromagnetic bandgaps, which can be used in gap-waveguide technology [21], filters [22], and to avoid leakage in flanges [23]. Here, we show two new advantages of glide symmetry. On the one hand, it closes the stopband between the first and second mode propagating below the hollow-waveguide cutoff frequency. On the other hand, having interleaved corrugations makes the propagation of low-dispersive forward modes possible, as well as the control of the stopband between the first and the second mode. These two properties are of interest for producing compact waveguide components, such as filters and phase shifters. These filters can be used in communications to avoid interferences between different frequency bands, and phase shifters can be used to control the radiation of aperture antennas. Aperture antennas based on waveguides also find application in radar systems.

In order to fundamentally understand the operation of the double-sided corrugated waveguides, we make use of the Multi-Modal Transmission-Matrix Method (MMTMM) [24]. This method has been used to characterize the propagation properties of periodic structures [25,26], including glide symmetry [27]. Its main advantage is that it permits accurate calculation of the attenuation constant, including effects due to radiation, dissipation losses, and electromagnetic bandgaps [28]. Moreover, with this method, we can evaluate the port modes that contribute to each propagating mode, and we can simulate any arbitrary configuration of corrugations.

## 2. Parametric Study of the Structure

**Figure 1:**
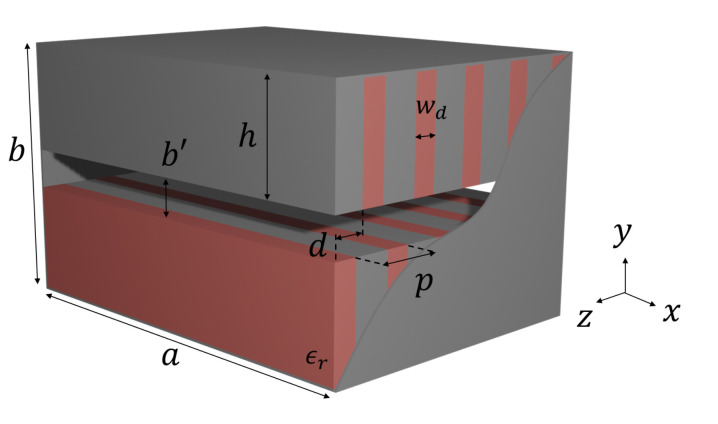
Rectangular metallic waveguide (a×b) with periodic corrugations (period *p*) on both the top and bottom sidewalls. The height of the corrugations is *h* ($b=2h+b^{\prime }$). Red regions stand for dielectrics (thickness $w_{d}$) and gray ones for metal (the metallic corrugation thickness is $p-w_{d}$). The top and bottom corrugations in this figure show a shift *d* between them.

A rectangular waveguide with dielectric-filled periodic corrugations on its bottom sidewall was proposed and studied in [8,9]. In the present work, we study a waveguide with corrugations on both the top and bottom sidewalls, which are misaligned at distance *d*, as illustrated in Figure 1.

**Figure 2:**
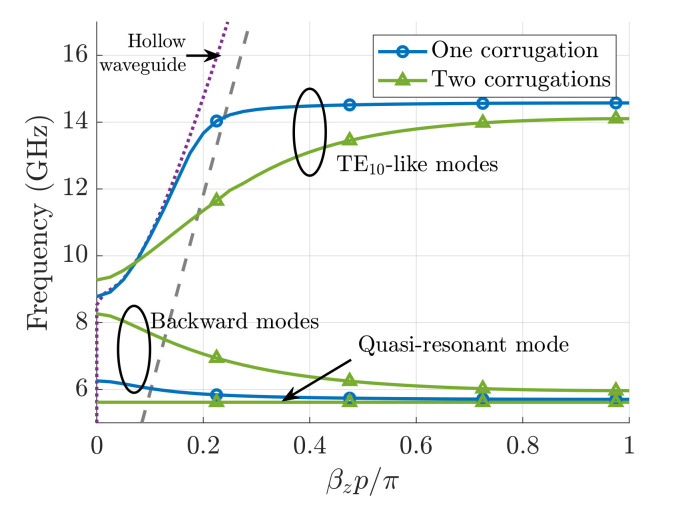
Dispersion diagrams ($\beta_{z}p$ is the phase shift) of corrugated waveguides loaded with dielectrics in the bottom sidewall and on both the bottom and upper sidewalls (mirror structure). The structure parameters are as follows: a=17 mm, b=10.16 mm, h=4.9 mm, p=2.54 mm, $w_{d}=1.016$ mm, and $\epsilon_{r}=10$. In the present case, there is no shift between top and bottom corrugations, namely d=0. The grey dashed line corresponds to the line of light and the purple dotted line to the dispersion diagram of a hollow waveguide with the same dimensions.

An example of the dispersion diagrams for these two types of waveguides are shown in Figure 2 (see the caption for their specific dimensions and dielectric permittivity).

The data shown in the figure have been obtained with the help of the *CST* Eigenmode solver. The boundary conditions in both *x* and *y* directions are set to Perfect Electric Conductor (PEC). As reported in [8] and observed in Figure 2, the presence of corrugations in only one sidewall gives rise to the existence of a backward mode below the cutoff frequency of the housing hollow waveguide (∼8.7 GHz). This mode is observed to be narrowband (from 5.7 GHz to 6.3 GHz) and very dispersive, after which a stopband extends until the cutoff frequency of the hollow waveguide. At this frequency, a Bloch TE${}_{10}$-like mode (that is, a mode with the same *x*-variation as the standard TE${}_{10}$ of the hollow waveguide) starts propagating up to 14.6 GHz, where a second stopband appears. In the so-called *mirror* waveguide (d=0), with corrugations on both top and bottom sidewalls, first we can observe a resonant mode (almost-horizontal green line) followed by a similar backward mode that now extends until the onset of the hollow-waveguide TE${}_{10}$ mode. The Bloch TE${}_{10}$-like mode of the mirror structure propagates now in the band 9.3–14.3 GHz, although it is less dispersive. Although small stopbands appear between the first and the second modes and between the second and the third modes, its first propagation band is significantly broader than the one of the single-sided corrugated waveguide. This is a clear advantage of the mirror corrugated waveguides in terms of compactness. In this example, the waveguide has a width a=17mm, and it allows for propagation from 5.7 to 14.3 GHz. A hollow waveguide with $f_{c}=5.7$ GHz has, in contrast, a width a=26.4 mm.

### 2.1. Effect of Breaking the Symmetry

**Figure 3:**
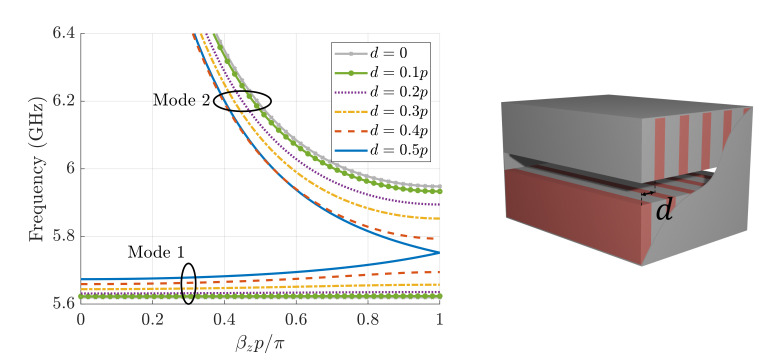
Dispersion diagram of the first two modes for different values of *d* of a corrugated waveguide with parameters: a=17 mm, b=10.16 mm, h=4.9 mm, p=2.54 mm, $w_{d}=1.016$ mm, and $\epsilon_{r}=10$.

In Figure 3, we illustrate the dispersion diagrams of the first two modes for different values of *d*. When d=0, the structure is mirrored, and when d=0.5p, the structure possesses glide symmetry. The first mode is a resonant mode (almost-horizontal lines), which beyond a stopband region gives rise to a second backward mode. When the corrugations are mirrored, the stopband appearing between the modes extends from 5.6 to 5.95 GHz. This stopband becomes narrower as the value of *d* increases, and it completely disappears for the glide-symmetric case for d=p/2, with both modes meeting at $\beta_{z}p=\pi$.

**Figure 4:**
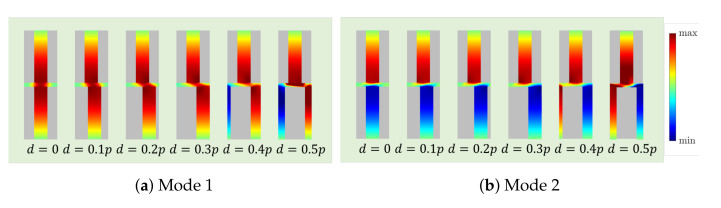
Plot of the amplitude of $E_{z}$-field inside the corrugations for the first two modes for different values of *d* at $\beta_{z}p=\pi$. The waveguide parameters are a=17 mm, b=10.16 mm, h=4.9 mm, p=2.54 mm, $w_{d}=1.016$ mm, and $\epsilon_{r}=10$.

Following the rationale in [13], in order to better understand this behavior, the amplitude of the *z*-component of the electric field when $\beta_{z}p=\pi$ is shown in Figure 4 for different values of *d*.

For the case d=0, there is a horizontal symmetry plane in the middle of the waveguide, so the modes can be classified based on their PEC or PMC symmetry plane. For example, the first mode has a PMC symmetry plane since the electric field in both top and bottom corrugations have the same direction, while the second one has a PEC symmetry plane. When the shift between the corrugations starts increasing, the plane of symmetry disappears, although the modes still have quasi-PEC/PMC symmetry for low values of *d*. This global quasi-symmetry completely disappears when the corrugations are glide-symmetric (d=0.5p), as discussed in [27]. In this situation, this middle plane of symmetry behaves as a PEC only for even harmonics and as a PMC only for odd ones, thus giving rise to the specific spatial profiles for modes 1 and 2 shown in Figure 4. Although the direction of $E_{z}$ is opposite in the bottom corrugations for modes 1 and 2, the global spatial profile of both modes is exactly the same in both cases. This fact is clearly in agreement with the well-known characteristic of glide-symmetric structures for which the effective period of the structure becomes p/2 (more precisely, the modes are eigenmodes of the glide operator mapping one corrugation into its glide counterpart) [12,27]. This fact additionally explains that both modes have to meet at $\beta_{z}p=\pi$ in Figure 3. It should be noted that, in this glide-symmetric case, mode 2 is no longer backward since this mode is just a continuation of mode 1, so its corresponding Poynting vector has the same direction as the phase advances. This shows a clear advantage of glide symmetry over the mirror version since the propagation is possible in a continuous frequency band.

Although only one mode propagates below cutoff in the glide-symmetric case, we will continue by discussing two modes in all cases for convenience.

### 2.2. Effect of Corrugations Height

**Figure 5:**
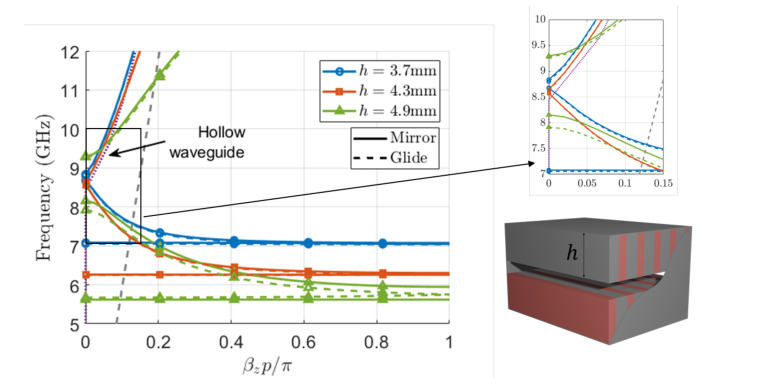
Dispersion diagram for different heights of top and bottom corrugations in a waveguide with dimensions a=17 mm, b=10.16 mm, p=2.54 mm, $w_{d}=1.016$ mm, and $\epsilon_{r}=10$. Solid lines represent the mirror structures and dashed lines represent the glide-symmetric structures. The grey dashed line corresponds to the line of light and the purple dotted line to the dispersion diagram of a hollow waveguide with the same dimensions.

In Figure 5, we illustrate the dispersion diagrams of the first three modes for different heights of the corrugations, both for the mirror and glide-symmetric cases. In the case of h=3.7 mm, we can observe that the dispersion characteristics of the mirror and glide cases are similar.

In this situation $b^{\prime }/b\approx 0.27$, which means that the bottom and top corrugations are not electrically close. The interaction between the corrugations is then expected to be weak, thus making the differences between the two cases quite small. It can be observed that the two first modes propagate in the band 7–8.7 GHz. At 8.8 GHz, the third mode, which is the TE${}_{10}$-like mode, starts to propagate. When the value of *h* increases to 4.3 mm ($b^{\prime }/b\;\approx \;0.15$, as in Figure 2), the similitude between the mirror and glide cases is still observed, and the band of propagation of the first and second modes increases (6.2–8.6 GHz). Finally, when the distance between the top and bottom corrugations is 0.36mm ($b^{\prime }/b\;\approx \;0.035$, as in Figure 3), the differences between the mirror and glide cases are more noticeable. In the mirror case, modes 1 and 2 propagate in the band 5.7–8.1 GHz having a stopband between the second and third modes from 8.1 to 9.2 GHz. Consequently, the third mode starts propagating above the cutoff frequency of the hollow waveguide. In the glide case, as already commented, the disappearance of the stopband can be observed between the first and second modes, as well as the widening of the stopband between the second and third modes.

### 2.3. Effect of the Permittivity

**Figure 6:**
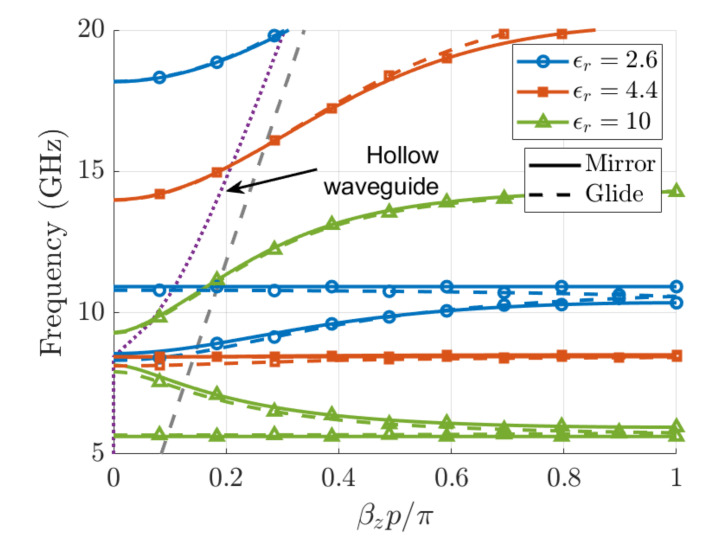
Dispersion diagram for different values of $\epsilon_{r}$ of a corrugated waveguide with parameters: a=17 mm, b=10.16 mm, h=4.9 mm, p=2.54 mm, and $w_{d}=1.016$ mm. Solid lines represent mirror structures and dashed lines represent glide-symmetric structures. The grey dashed line corresponds to the line of light and the purple dotted line to the dispersion diagram of a hollow waveguide with the same dimensions.

The dispersion diagrams for different permittivities when h=4.9mm ($b^{\prime }/b\approx 0.035$) are plotted in Figure 6. A first interesting observation comes from the different behavior that the first and second modes have as the permittivity of the filling dielectric varies. In particular, for $\epsilon_{r}=10$, it was found that the second mode was backward and appears below the cutoff frequency of the housing waveguide (8.6 GHz). However, for $\epsilon_{r}=2.6$, the second mode was forward, and its onset appears above the hollow-waveguide cutoff. The existence of backward bands below the housing waveguide cutoff was already reported in [2,4,5,8], where it was discussed that some periodic structures were able to create frequency bands where both the effective permeability and permittivity are simultaneously negative, thus giving rise to left-handed metamaterial-type guided-wave structures. It should be reminded that below the cutoff of the hollow waveguide, the TE${}_{10}$ has a capacitive nature; hence, it is equivalent to a medium with effective negative permittivity [1,2]. This situation of double negative effective permeability, and permittivity is what we find for $\epsilon_{r}=10$. For frequencies greater than the hollow-waveguide cutoff, the equivalent effective permittivity of the mode is positive; thus, we expect forward propagation if the effective permeability is also positive and rejection bands if this permeability is negative [29]. The presence of backward modes and stopbands allow us to estimate where a negative-effective-permeability (NEP band) is achieved. Namely, for $\epsilon_{r}=10$, the NEP is found to extend up to 9.3 GHz, observing a short stopband from 8 to 9.3 GHz, which is continued by a forward propagative band. This band ends at 14.3 GHz, where another NEP band starts and gives rise to another rejection band.

When the permittivity of the filling dielectric decreases, the first NEP region moves up in frequency. If the lower bound of this frequency band is no longer below the cutoff frequency of the hollow-waveguide, no backward modes are expected at low frequencies. This situation is what we find for $\epsilon_{r}=2.6$, where the first band is forward at the range from 8.4 to 10.8 GHz. At this last frequency, a rejection band extends up to 18.2 GHz, coinciding with the presence of the first NEP region. An intermediate and interesting situation is found for $\epsilon_{r}=4.4$, where the lower frequency bound of the NEP regions almost coincides with the cutoff frequency of the TE${}_{10}$ mode. This situation rise place to a resonant mode that appears in the vicinity of the cutoff frequency. When this NEP band ends at 14 GHz, a forward propagative band starts.

## 3. Multi-Mode Transfer Matrix Method (MMTMM)

The MMTMM is an efficient simulation-assisted method able to compute not only the phase shift but also the attenuation factor of arbitrary periodic structures [24,25,26,27,28]. As any periodic structure is a concatenated system of unit cells, the full structure can be fully characterized by using the ABCD (or transfer) matrix, [T], of the corresponding unit cell. This unit cell is simulated by using any commercial or in-house full-wave software in order to obtain the *multimodal* scattering matrix, which is later transformed to a multimodal transfer matrix. In this work, the frequency domain solver of the commercial software *CST* has been used for this purpose. The use of powerful full-wave simulators makes the treatment of very complex unit cells possible (arbitrary geometry, as well as a great variety of materials). Different practical questions associated with the application of the MMTMM have been discussed in [28]. In particular, for the present structure with metallic corrugations wider than the dielectrics, the input/output ports of the unit cells in the simulation are conveniently chosen to be homogeneous metallic-waveguide sections. This choice greatly simplifies the analysis of the full-wave simulator and the following post-processing involved in the MMTMM by ensuring that no hybrid modes are excited in the input/output ports of the unit cell. After computing the transmission matrix of the unit cell (period *p*), the dispersion diagram for one-dimensional periodic structures is calculated by solving the following eigenproblem:

(1) $$\left[\begin{array}{c} V_{2}\\ I_{2} \end{array}\right]=\left[T\right]\left[\begin{array}{c} V_{1}\\ I_{1} \end{array}\right]=e^{-j\;k_{z}p}\left[\begin{array}{c} V_{1}\\ I_{1} \end{array}\right]$$

where $V_{i}$ and $I_{i}$ are the input/output voltage and current vectors, respectively, and $k_{z}$ is the Bloch modal wavenumber. As observed in (1), the phase factor $e^{-j\;k_{z}p}$ is an eigenvalue of the ABCD matrix. Here, $k_{z}$ is defined as $k_{z}=\beta_{z}-j\alpha_{z}$, where $\beta_{z}$ is the phase constant, and $\alpha_{z}$ denotes the attenuation constant.

### 3.1. Numerical Results

**Figure 7:**
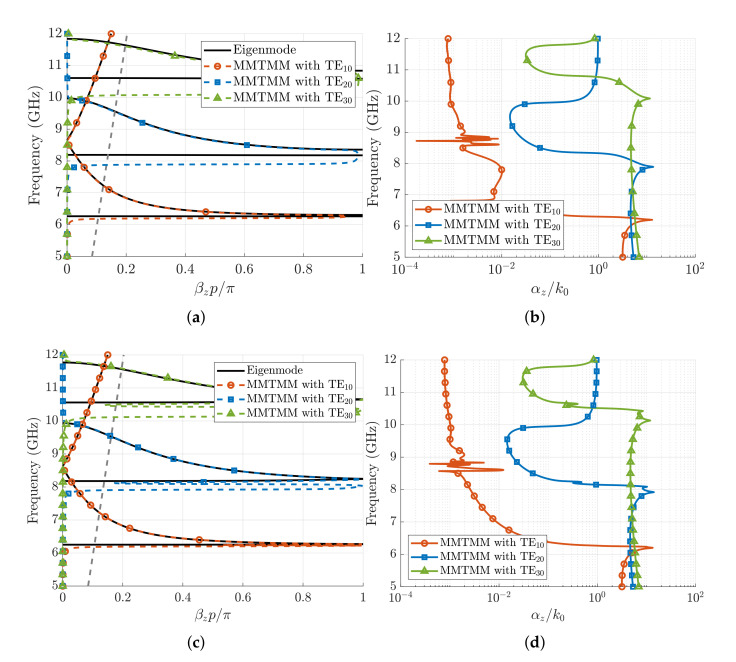
Dispersion diagram (phase shift and normalized attenuation constant) of the TE${}_{10}$, TE${}_{20}$, and TE${}_{30}$ modes computed with the MMTMM. Waveguide parameters are as follows: a=17 mm, b=10.16 mm, h=4.3 mm, p=2.54 mm, $w_{d}=1.016$ mm, and $\epsilon_{r}=10$ with a tangent of losses tanδ=0.002. Black lines correspond to the phase-shift results of the Eigenmode solver in CST for comparison. (**a**,**b**): Mirror waveguide. (**c**,**d**): Glide-symmetric waveguide. The grey dashed line corresponds to the line of light.

Figure 7 shows the results provided by the MMTMM for the first three Bloch modes of the structure, with the dimensions and permittivity shown in the caption, for both the mirror and glide-symmetric waveguides when material losses are considered in the filling dielectric. The results were computed when three input/output port modes (TE${}_{10}$, TE${}_{20}$, and TE${}_{30}$) are simultaneously considered in the transfer matrix, as well as when the transfer matrix only involves each port mode separately. No significant difference was found in these computations since no coupling between the chosen port modes is appreciated. For both the mirror and glide-symmetric waveguides, when the input/output waveguide port mode is the TE${}_{10}$, the application of the MMTMM gives us the dispersion diagrams of the first two modes that propagate below the hollow-waveguide cutoff frequency, with the third mode being analogous to TE${}_{10}$ of the hollow waveguide (this third mode is the one starting at about 8.6 GHz). A good agreement is found between the phase-shift data provided by the present method and the ones computed with the *CST* Eigenmode solver. However, this solver does not directly provide the attenuation constant of these modes either if it is caused by material losses or rejection within the stopband. In both mirror and glide cases, the attenuation constant calculated with the MMTMM shows a maximum value of $\alpha_{z}/k_{0}=13.2$ at 6.2 GHz where the resonance happens. From there, the normalized attenuation rapidly decreases until $10^{-3}$ since the mode propagates in all the band. In the range 8.5–9 GHz, where the second and third mode connect, the numerical value of the normalized attenuation is found to be noisy because of the required operations with ill-conditioned matrices.

When the TE${}_{20}$ port mode is the operating mode in the MMTMM, it provides the dispersion diagram of the modes with two transversal variations along the *x*-direction. These modes appear as the second resonant and backward modes in the phase-shift diagrams of Figure 7, in the frequency band 8.5–9.9 GHz. For the glide-symmetric case, a double resonance is found before the mode starts to propagate. Observing the attenuation constant of this mode in its propagating region, we find that the attenuation of this TE${}_{20}$-like Bloch mode is significantly higher than the one for the TE${}_{10}$-like Bloch mode. The TE${}_{20}$-like Bloch mode reaches its maximum value at 7.9 GHz where $\alpha_{z}/k_{0}=13.55$ in the mirror case, and $\alpha_{z}/k_{0}=14.2$ in the glide case. At 9.9 GHz, the attenuation constant grows again, however, the rejection level in this stopband is not as high as before ($\alpha_{z}/k_{0}\approx 1$).

The introduction of the TE${}_{30}$ port mode in the MMTMM solves the dispersion diagram of the last set of modes, which propagate in the band from 10.7 to 11.9 GHz. Again, the MMTMM solution has double resonance in the glide-symmetric case instead of the single one observed for the mirror case. The attenuation constant reaches its maximum at 10 GHz, with similar values as in the previous TE${}_{20}$ case. After that frequency, the level of attenuation decreases to 0.003 within the band where these modes propagate.

In the mirror case, we can observe some discrepancies between the MMTMM results and the ones provided by the CST Eigenmode solver for the resonant modes. As later discussed, the reason is that more than one single mode is required for the MMTMM to converge in these regions.

### 3.2. Convergence Study

A relevant issue concerning the application of the MMTMM is to ensure that the numerical values of the Bloch wavenumbers provided by the method do not significantly vary as the number of high-order modes in the input/output ports increased [27,28]; namely, we have to assure that a good level of convergence is achieved. In this section, we study the convergence of the first resonant mode and the second backward mode of both the mirror and glide structures. Since these modes are associated with the lowest possible transverse variation in the *x*-direction, waveguide-port modes with only one transverse variation in this direction (TE${}_{10}$, TE${}_{11}$, TM${}_{11}$, *…*) will be used to study the convergence of the method.

**Figure 8:**
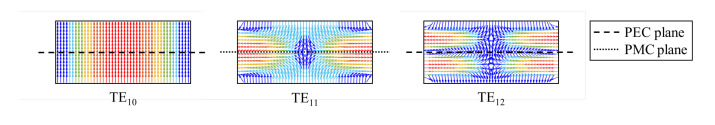
Spatial profiles of waveguide-port modes.

As previously stated in Section 2.1, the resonant mode (mode 1) in the mirror structure has a PMC symmetry plane in the *x* direction, while the backward one (mode 2) presents a PEC symmetry plane there. Hence, port modes with a PMC symmetry plane will contribute to the convergence of mode 1, and port modes with a PEC symmetry plane contribute to mode 2. In Figure 8, the spatial profiles of three different waveguide-port modes are shown. Modes TE${}_{10}$ and TE${}_{12}$ have a horizontal PEC symmetry plane, whereas the TE${}_{11}$ mode has a PMC symmetry plane. TM modes have the same symmetry plane as their TE counterparts, i.e., the TM${}_{11}$ mode has a PMC symmetry plane, and the TM${}_{12}$ mode has a PEC symmetry plane. Consequently, TE and TM modes will be added in pairs to study the convergence.

**Figure 9:**
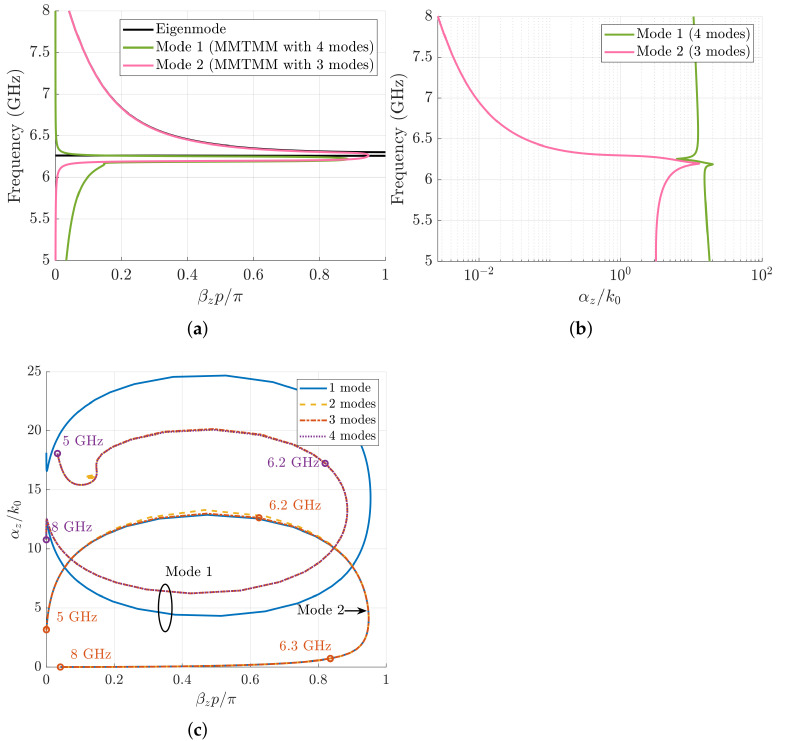
Dispersion diagram of the first two modes computed with the MMTMM for the mirror case. Waveguide parameters include the following: a=17 mm, b=10.16 mm, h=4.3 mm, p=2.54 mm, $w_{d}=1.016$ mm, and $\epsilon_{r}=10$ with a tangent of losses tanδ=0.002. Black lines correspond to the phase-shift results of the Eigenmode solver in CST for comparison. (**a**) Phase shift. (**b**) Attenuation constant. (**c**) Root loci of the first and second Bloch modes for different number of waveguide-port modes.

The converged values of the phase shift and attenuation constant obtained with the MMTMM for a mirror structure are plotted in Figure 9a,b, respectively. Four modes are employed to calculate mode 1 (TE${}_{11}$, TM${}_{11}$, TE${}_{13}$, and TM${}_{13}$) and three modes for mode 2 (TE${}_{10}$, TE${}_{12}$, and TM${}_{12}$). The phase shift of the resonant Bloch mode increases from 0 to 0.1 in the band below 6.2 GHz where the resonance happens. The attenuation constant has a high value in the lower frequencies, reaching its maximum of $\alpha_{z}/k_{0}=20$ at 6.2 GHz. From there, the attenuation decreases rapidly to $\alpha_{z}/k_{0}=6$ at 6.3 GHz where it starts growing again. If the materials were ideal, the attenuation constant would decrease to zero in a very narrow frequency band (somehow similar to a δ function). However, small values of material losses seem to give rise to high attenuation levels, which in practice would preclude the effective excitation of the resonant mode. For the second odd Bloch mode, it is found that its phase shift is zero for lower frequencies and starts growing to $\beta_{z}p/\pi =1$ at about 6 GHz. From 6.3 GHz, the MMTMM agrees well with the Eigenmode solver. Its attenuation, on the other hand, grows in the lower frequencies up to 6.2 GHz, where it reaches its maximum value ($\alpha_{z}/k_{0}=13$). From there, the attenuation constant decreases to $10^{-3}$ (as already mentioned in the previous section). The convergence of each Bloch mode is analyzed with the root loci shown in Figure 9c. The first mode converges with two port modes (TE${}_{11}$ and TM${}_{11}$), whereas the second Bloch mode converges with only one port mode (TE${}_{10}$). The different root loci have a sort of elliptical shape due to the material losses added. As mentioned before, the even mode is a complex mode with high values of attenuation in all the studied frequency band. Nevertheless, the odd mode behaves as a propagative one with small losses above 6.3 GHz.

**Figure 10:**
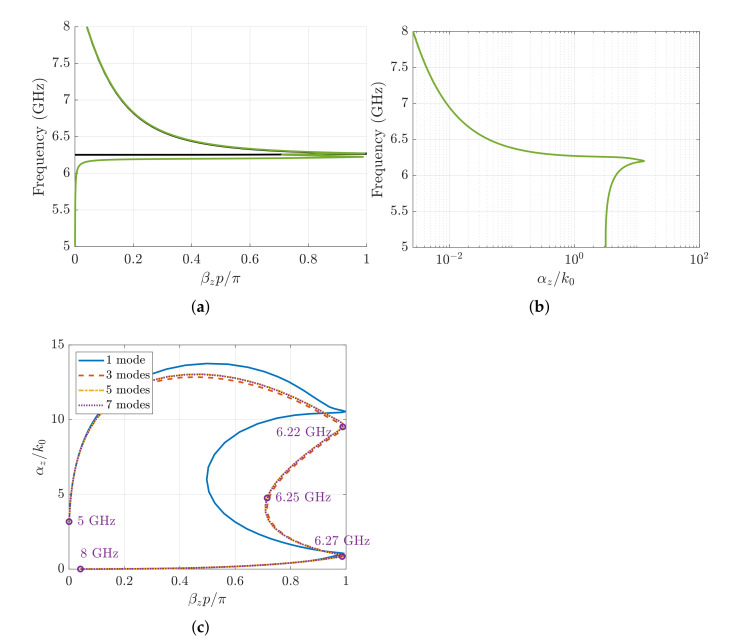
Dispersion diagram of the first two modes computed with the MMTMM for the glide-symmetric case. Waveguide parameters include the following: a=17 mm, b=10.16 mm, h=4.3 mm, p=2.54 mm, $w_{d}=1.016$ mm, and $\epsilon_{r}=10$ with a tangent of losses tanδ=0.002. Black lines correspond to the phase-shift results of the Eigenmode solver in CST for comparison. (**a**) Phase shift. (**b**) Attenuation constant. (**c**) Root loci for different number of modes.

In contrast to the mirror case, the Bloch modes of a glide-symmetric structure does not have global symmetry that makes difference of even and odd port modes. Actually, both sets of waveguide-port modes are required to account for the Bloch modes in glide structures [27]. In Figure 10a,b, the phase shift and attenuation constant retrieved by the MMTMM are plotted. These solutions have been calculated with the first seven modes (TE${}_{10}$, TE${}_{11}$, TM${}_{11}$, TE${}_{12}$, TM${}_{12}$, TE${}_{13}$, and TM${}_{13}$). At 6 GHz, a double resonance occurs, after which the backward mode starts propagating. The attenuation constant grows from 5 to 6.2 GHz, where its maximum value is reached ($\alpha_{z}/k_{0}=12.8$). This frequency point coincides with the first resonance. After that, the attenuation starts decreasing up to $10^{-3}$. Note that the resonant mode retrieved by the Eigenmode solver agrees with the second resonance of the MMTMM solution. The reason is that the Eigenmode solver can only calculate real modes, and the first resonance has a high imaginary part. Finally, in Figure 10c, the root loci for different number of modes is shown. The modes were added in pairs of TE and TM port modes as discussed before. A good convergence for this mode is found with the first three port modes (TE${}_{10}$, TE${}_{11}$, and TM${}_{11}$). The attenuation starts growing while the phase shift moves closer to 1. The frequencies where the two resonances occur (6.22 and 6.27 GHz) are also pointed out. Moreover, it can be observed that the mode starts behaving as a propagating one with small losses after the second resonance.

Here, it is interesting to note that the authors could not corroborate the values of the attenuation constants shown in Figure 9 and Figure 10 by means of a *CST* simulation of a truncated structure with several unit cells. The reason is that the frequency band studied in those figures is below the hollow waveguide’s cutoff frequency; thus, the solver cannot normalize the S-parameters with the correct impedance. This drawback does not appear when applying the MMTMM since the wavenumbers it provides do not depend on any normalized impedance [28].

## 4. Interleaved Corrugations

**Figure 11:**
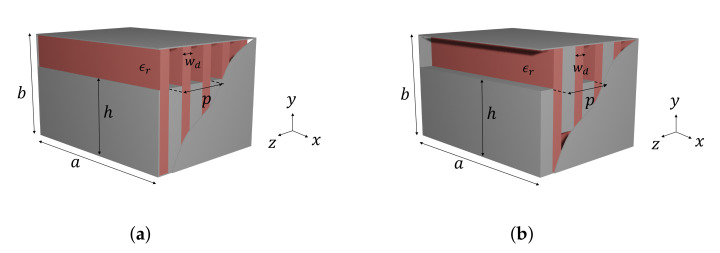
Rectangular waveguide with interleaved metallic and dielectric corrugations. (**a**) Conventional and (**b**) glide-symmetric structures.

In order to illustrate the potential advantages of glide symmetry, a new structure is studied in this section, as shown in Figure 11b.

It consists of a rectangular waveguide with glide-symmetric metallic corrugations for which its height is longer than half of the waveguide’s height (h>b/2). Between corrugations, a dielectric is placed covering the entire transversal section of the waveguide. The glide-symmetric structure is compared with its conventional counterpart, shown in Figure 11a, where all the metallic corrugations are placed in the bottom sidewall. The unit cell of the conventional structure is taken with two periods for a fair comparison with its glide-symmetric counterpart.

**Figure 12:**
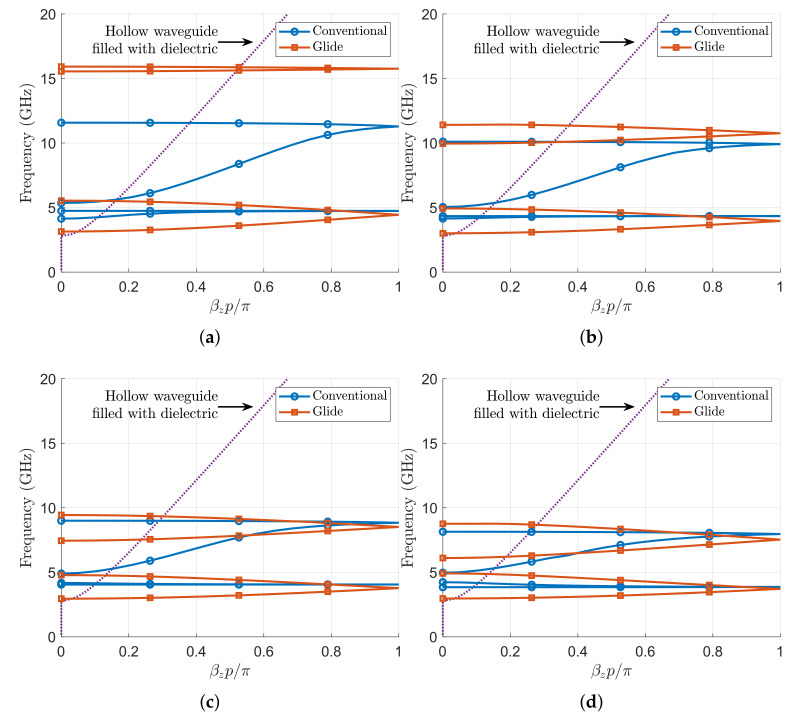
Dispersion diagram of a waveguide with interleaved corrugations computed with the Eigenmode solver. Waveguide parameters are as follows: a=17 mm, b=10.16 mm, p=5.08 mm, $w_{d}=1.016$ mm, and $\epsilon_{r}=10$. Purple dotted lines correspond to the phase-shift of the hollow waveguide filled with a dielectric with permittivity $\epsilon_{r}=10$. (**a**) h=6 mm. (**b**) h=7 mm. (**c**) h=8 mm. (**d**) h=9 mm.

In Figure 12, the dispersion diagrams of these two structures up to 20 GHz are plotted for different corrugation heights *h*. In the first case, with h=6 mm, the conventional structure allows the propagation of two forward modes: the first one in the band 4.1–4.7 GHz and a second one in the band 5.4–11.6 GHz. The glide-symmetric structure also allows the propagation of two forward modes, with significant differences with respect to the ones of the conventional structure. First, it can be observed that the modes of the glide-symmetric structure are less dispersive. The first mode starts propagating at the cutoff frequency (3 GHz) of the rectangular waveguide homogeneously filled with a dielectric of $\epsilon_{r}=10$ and continues propagating up to 5.5 GHz. A second relevant difference is observed in the stopband between the first and the second mode, which broadens considerably from 5.5 to 15.6 GHz.

When the corrugation’s height increases to 7 mm, the dispersion diagram plotted in Figure 12b shows that both modes in the conventional case narrow their propagating band; the first mode propagates between 4.1 and 4.3 GHz, and the second one propagates between 5 and 10 GHz. For the glide-symmetric case, it can be observed that its modes retain a lower dispersive nature. The first mode has the same operating band than the case of h=6 mm. The second mode, however, increases its bandwidth up to 1.4 GHz, while narrowing the stopband from 5.5 to 10 GHz.

For the case with h=8 mm shown in Figure 12c, in the conventional case, the propagating band of the first mode reduces to 4–4.1 GHz, and the second mode narrows its upper bound up to 9 GHz. Similarly, the glide-symmetric structure has a first mode that propagates in the same frequency band as the case of h=7 mm, but the frequency band of the second mode drops down to 7.5–9.4 GHz.

The evolution of the modes with increasing values of *h* reveals that the differences between the dispersion diagram of the conventional and the glide-symmetric structures tend to diminish. This trend is confirmed by looking at the dispersion diagram in Figure 12d, when the corrugations have a height of 9 mm. In this case, the first mode of the conventional structure propagates in the band between 3.8 and 4.1 GHz and the second mode between 5 and 8.1 GHz. The two passbands of the glide-symmetric structure now appear between 3 and 5 GHz and another between 6.1 and 8.8 GHz.

## 5. Conclusions

Here, we presented a rectangular waveguide with metallic periodic corrugations loaded with dielectrics on both top and bottom sidewalls. This structure allows for the propagation of a quasi-resonant mode and a backward mode below the cutoff frequency of the hollow waveguide. The backward mode is less dispersive and propagates in a broader bandwidth than the one previously found in a similar structure with corrugations in only one sidewall. The appearance of this below-cutoff low-dispersive propagating mode can be useful for waveguide miniaturization. The stopband that appears between the quasi-resonant and the backward modes is reduced as the top and bottom corrugations are misaligned, until it completely disappears in the glide-symmetric case. This specific observation is attributed to the already-reported fact of having both PEC (for even harmonics) and PMC (for odd harmonics) symmetry planes in glide-symmetric scenarios.

Extensive parametric studies have been carried out. In particular, we have studied the effect of the heights of the corrugations and the dielectric permittivity. The height of the corrugation is found to have an impact on the bandwidth of the backward mode, whereas the value of permittivity mostly influences the frequency band where the negative-effective-permeability zone appears. The multi-modal transmission-matrix method has been used in this work to estimate the losses in the structure. Moreover, a study on the convergence of the first two Bloch modes of the periodic structure has shown that the method requires at least the inclusions of the first three port modes with one variation in the *x* direction.

Finally, we analyzed another version of the structure where the corrugations are interleaved. For this case, the glide-symmetric version allows for the propagation of forward modes that are less dispersive in a broader band than its conventional counterpart. Moreover, the stopband between the first two passbands can also be enhanced. These characteristics clearly show that glide-symmetric configurations can provide novel and useful practical capabilities to periodic structures. For example, these properties are useful for waveguide components (such as filters and phase shifters) and aperture antennas, which find application in communications, sensors, and surveillance.

## Data Availability

The data that support the findings of this study are available from the corresponding author upon reasonable request.
